# Lump at the back of my tongue. The lingual thyroid–A case report and narrative review of literature

**DOI:** 10.1002/ccr3.4160

**Published:** 2021-05-06

**Authors:** Joe Jabbour, Nelson Agostinho, Harinder K. Bains, Peter Earls, Louise Killen, Ruwan Perera, Ranga Sirigiri, Hayder Ridha

**Affiliations:** ^1^ Department of Otolaryngology Dubbo Base Hospital Dubbo NSW Australia; ^2^ Department of General Surgery Dubbo NSW Australia; ^3^ Department of Anatomical Pathology Darlinghurst NSW Australia

**Keywords:** airway, ectopic, thyroid, thyroidectomy, transoral robotic surgery

## Abstract

Although the ectopic thyroid in adults is rarely symptomatic, biochemistry and imaging workup are essential. Treatment modality of choice is dependent on patient factors, institution factors and surgeon factors. The mainstay treatment involves hormone suppression treatment with exogenous thyroid hormone. If medical management is unsuccessful, surgical excision requires an experienced team including an anaesthetist and otolaryngologist. Anaesthetic considerations are important because intubation may be a potentially difficult procedure secondary to potential serious obstruction of the upper airway. We present a case report and narrative review of the literature regarding lingual thyroid workup and management.

## Graphical abstract

The lingual thyroid may be managed surgically if medical treatment is unsuccessful. The lesion is removed en bloc usually via transoral robotic surgery and is demonstrated in figure 3 with a cut surface revealing a multi‐located calcified cyst.

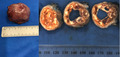

## 1 INTRODUCTION

A lingual thyroid (LT) is a rare anomaly of functioning ectopic thyroid tissue found at the base of the tongue and represents the most common location of functioning ectopic thyroid tissue.^1^ The incidence of LT is approximately 1 in 100 000 cases. LT is four times more common in females than in males and often manifests in the second decade of life.^1^, ^2^ The diagnosis of LT is suspected clinically and confirmed with radionucleotide scanning (Technetium pertechnetate thyroid scan).^1^

## 2 CASE REPORT

A 68‐year‐old male patient presented to a rural emergency department with a 48‐hour history of right upper quadrant pain associated with nausea and vomiting on the background of poorly controlled insulin‐dependent diabetes, hypothyroidism, GORD, and hypertension. Regular pharmacotherapy included insulin, pantoprazole, and thyroxine. Investigation revealed cholecystitis with choledocholithiasis. An ERCP was initially performed to relieve the biliary tree obstruction followed by an interval cholecystectomy. At induction for cholecystectomy, the anesthetic team encountered a difficult airway due to a firm mass at the base of the tongue. An airway was eventually established allowing completion of a laparoscopic cholecystectomy without issue. The patient was routinely extubated and had an uneventful postoperative recovery.

**FIGURE 1:**
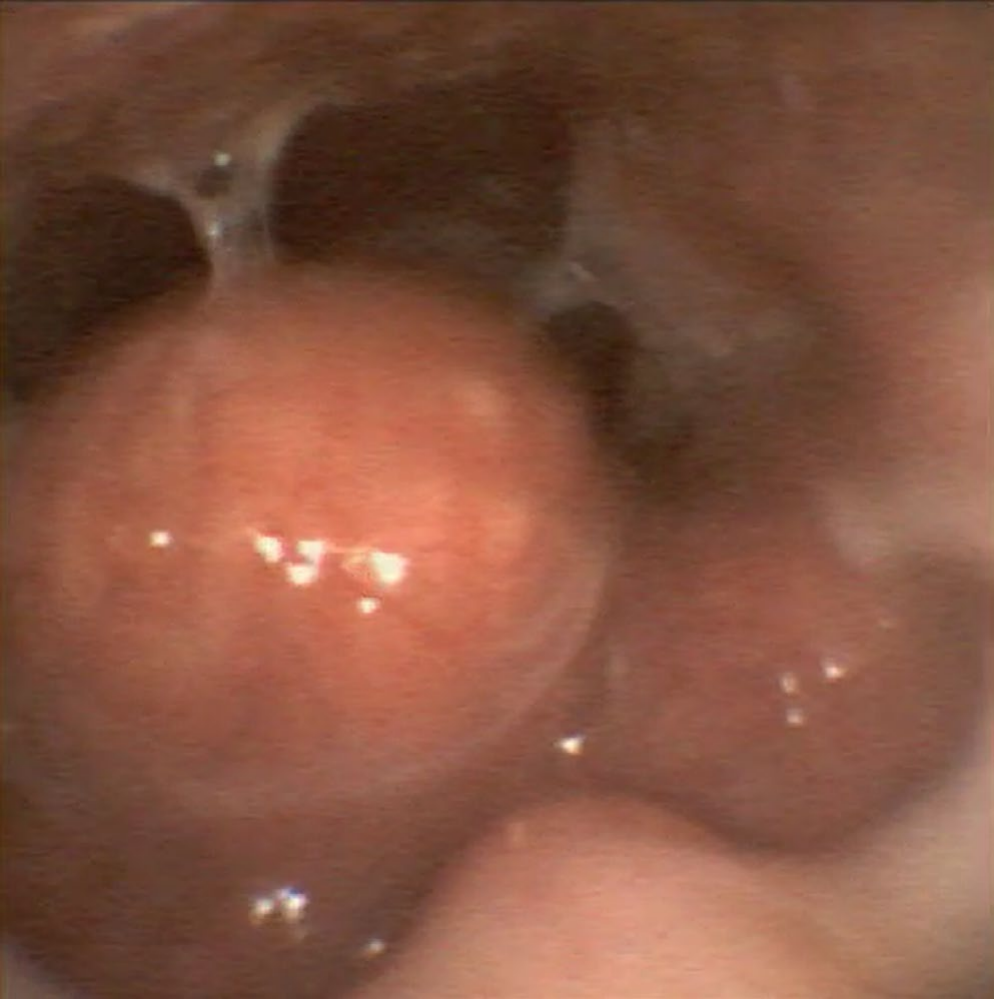
Nasoendoscopy of lingual thyroid: Round tongue base mass obstructing the laryngeal inlet

**FIGURE 2:**
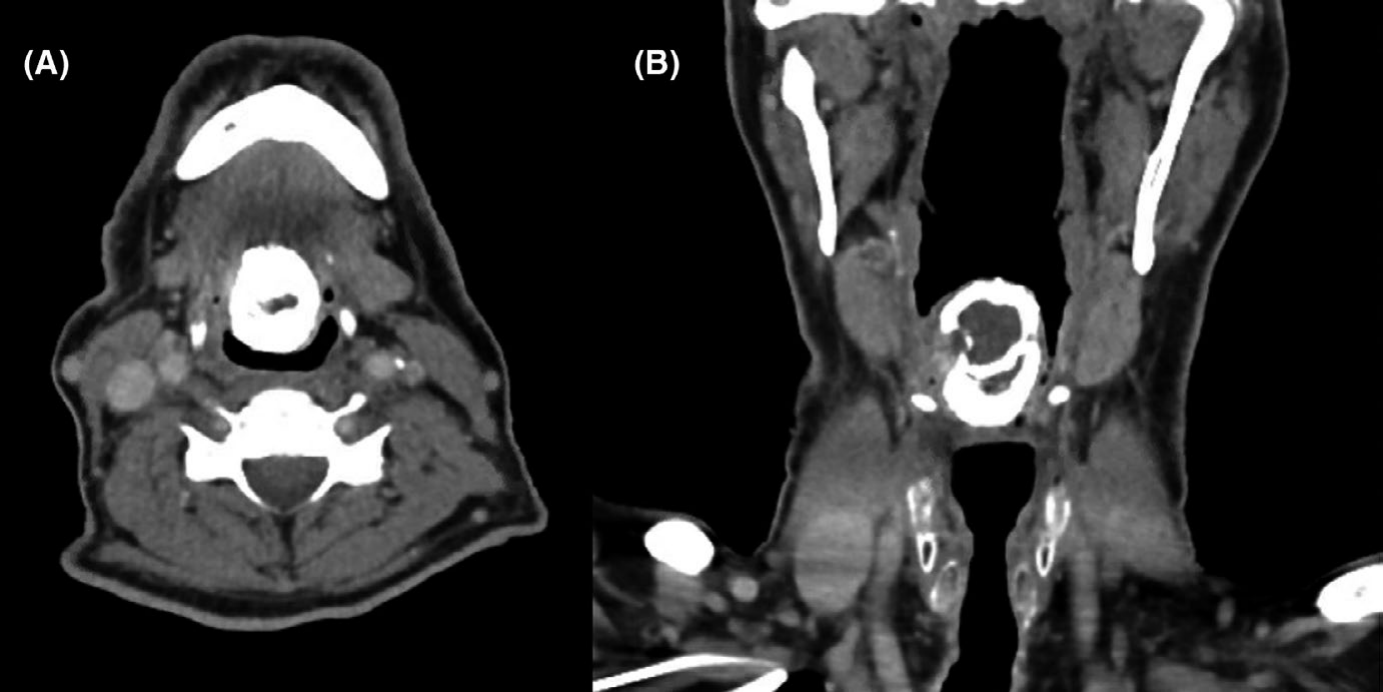
CT neck soft tissues with IV contrast: A, Axial view of rim‐calcified mass at midline of tongue base measuring 30 × 32 mm in anteroposterior diameter; B, Coronal view of rim‐calcified mass at tongue base measuring 40 mm in lengt

**FIGURE 3:**
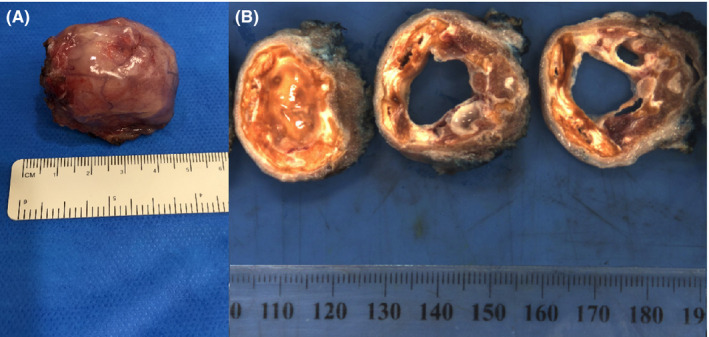
A, Macroscopic lingual thyroid specimen; B, The surgical margin is inked blue. Cut surface shows a multi‐loculated calcified cyst 30 × 25 × 40 mm

**FIGURE 4:**
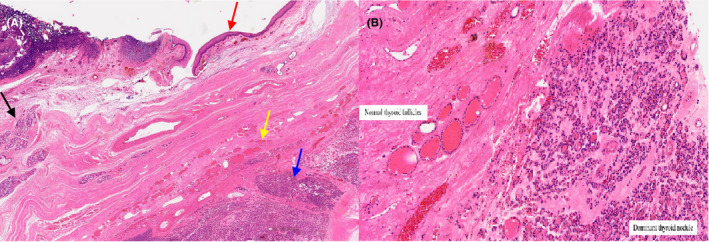
Microscopic histopathology of lingual thyroid specimen: A, Low magnification demonstrates oral type squamous mucosa (Red arrow), with subepithelial stroma, minor salivary glands (Black arrow), normal thyroid follicles (Yellow arrow), and a dominant thyroid nodule (Blue arrow); B, High magnification demonstrates thyroid tissue with features of nodule hyperplasia with a dominant (adenomatoid) partly calcified nodule within the subepithelial stroma and skeletal muscle

During the postoperative period, the encountered base of tongue mass was investigated. The patient revealed a history of dysphagia and imminent airway obstruction requiring an emergency tracheostomy 15 years prior to this admission. Nasoendoscopy revealed a large base of tongue lesion with associated displacement of the epiglottis (Figure 1). An ultrasound of the neck revealed no tissue in the thyroid bed with calcified structure at the tongue base. A computer tomography (CT) of the neck was then performed that supported the finding of no tissue in the thyroid bed and a calcified mass at the base of the tongue with a volume of 40 cc measuring 29 mm (anteroposterior) and 26 mm (transverse) (Figure 2). Biochemical investigations revealed that the patient was euthyroid. The patient was eventually accepted for a transoral robotic surgery (TORS) at a tertiary center. This was performed without complication and the lingual thyroid mass sent for histopathology (Figure 3). Histopathology of the lesion revealed oral type squamous mucosa with subepithelial stroma, thyroid tissue, minor salivary glands, and skeletal muscle (Figure 4).

## 3 DISCUSSION

The mainstay of nonsurgical treatment involves hormone suppression treatment (HST) with exogenous thyroid hormone to induce atrophy of the gland by causing negative feedback in the pituitary/thyroid axis.^3^, ^4^ Patients have regular follow‐up for clinical examination and thyroid function tests. The literature suggests treatment effectiveness in 61% of patients.^2^ Surgical excision of the LT is indicated when patients receiving HST remain symptomatic. Another option for patients who fail HST or are not candidates for surgical excision is radioactive ablation with I‐131.^5^ If the patient is euthyroid and asymptomatic, then treatment may not be necessary.

### 3.1 Surgical: Excision alone or excision with autotransplantation into muscle

Surgery for LT can be offered for patients with airway compromise, dyspnea, dysphagia, speech impairment, globus pharyngeus, or OSA.^6^ Technetium 99 (99mTc) and neck ultrasound will determine whether surgical excision alone is required or excision with autotransplantation into muscle. The three main approaches to surgical excision of lingual thyroid are as follows: transoral, transmandibular, and lateral pharyngotomy.^7^ Open surgery has been associated with increased morbidity and prolonged hospitalization. Thus, CO2 laser, electrocautery assisted resection with rigid endoscope and operating microscope and suspension laryngoscopy have been attempted,^8^ although the aforementioned approaches are limited by safe visibility and difficulty in manipulation rendering resection more difficult.

The lateral pharyngotomy approach involves accessing the lingual thyroid through a cervicotomy incision in the neck. This technique is mandible‐sparing, although important anatomical obstacles are countered: the lingual artery, hypoglossal nerve, superficial laryngeal nerve, and the superior thyroid artery. Risks with this technique include development of pharyngocutaneous fistula formation and skin scar.^8^

Transoral robotic surgery (TORS) is a safe and feasible minimally invasive approach for excision of the lingual thyroid with larger three‐dimensional point of view and easier manipulation due to freedom of motion of robotic instruments.^9^, ^10^ The predominant risk with transoral robotic lingual thyroid resection is lingual artery injury. This injury can be prevented by preoperative imaging methods and careful dissection with knowledge of anatomy.^10^ The anatomic relationship may be effected by mass effect, and thus, the routine course of the lingual artery may vary.^10^ Absolute local contraindication is limited mouth opening or trismus.

The transmandibular approach for excision of lingual thyroid provides wide exposure of tongue and reduces the need for a tracheostomy. This approach involves lip split mandibulotomy, dissection of mylohyoid muscles to reach the base of the tongue and exposure of lingual mass. The tumor is dissected out and excised. The osteotomized mandible is plated with primary closure of superficial layers. Risks associated with the transmandibular approach include lip hypoesthesia, facial disfigurement, malocclusion of teeth, and temporomandibular joint pain. Regardless of the surgical approach, lifelong exogenous thyroid hormone replacement is required.

### 3.2 Anesthetic considerations

Securing the airway is a crucial component of LT surgical management. In children inhalation induction of general anesthesia is used to ensure spontaneous ventilation and avoid complications of asleep intubation and inability to secure the airway. A flexible video laryngoscopy, a difficult airway trolley, and a tracheostomy tray must be step up at the bedside. Paralytic agents of skeletal muscle should be avoided to prevent cessation of spontaneous ventilation and increased risk of a surgical airway. The trachea in children can usually be anesthetized with a deep inhalation agent. Capnography monitoring of exhaled gas analysis is useful in determining the depth of anesthesia. Fiberoptic awake nasotracheal intubation is the preferred method of securing the airway to allow increased visibility and maneuverability for the surgeon during excision of the lingual thyroid. The type of endotracheal tube used (cuffed or uncuffed) and size depend on the patient's age. However, to prevent subglottic stenosis, there needs to be an air leak at 20cm H2O. Securing the airway in an adult involves high‐flow nasal prong oxygenation, fiberoptic nasoendoscopic application of topical anesthesia with light sedation and subsequent nasotracheal intubation while awake.

## 4 CONCLUSION

Ectopic thyroid is a rare entity more common in females. Symptomatic patients require careful workup including diagnostic biochemistry and imaging to confirm normal thyroid‐producing tissue. Initial management needs to include anesthetic considerations and securing an imminent airway obstruction. Treatment modality of choice is dependent on patient factors, institution factors, and surgeon factors.

## AUTHOR CONTRIBUTIONS

JJ, NA & HB contributed to literature review, analysing the data and writing the manuscript. PE, LK, RP, RS and HR contributed to writing the manuscript and reviewing the content.

## CONFLICT OF INTEREST

There are no conflicts of interest to declare and no financial disclosure.

## ETHICAL APPROVAL

The authors assert that all procedures contributing to this work comply with the ethical standards of the relevant national and institutional guidelines on human experimentation and with the Helsinki Declaration of 1975, as revised in 2008.

## Data Availability

Data sharing not applicable to this article as no datasets were generated or analyzed during the current study.

## References

[1] Kumar SS , Kumar DMS , Thirunavukuarasu R . Lingual thyroid‐conservative management or surgery? A case report. Indian J Surg. 2013;75(suppl 1):118‐119. 24426535 10.1007/s12262-012-0518-4 PMC3693310

[2] Kamat MR , Kulkarni JN , Desai PB , Jussawalla DJ . Lingual thyroid: a review of 12 cases. Br J Surg. 1979;66(8):537‐539. 486909 10.1002/bjs.1800660805

[3] Alharbi A . Lingual thyroid: a systematic review of hormonal suppression treatment. J Otolaryngol ENT Res. 2015;2(3):115‐118.

[4] Ueda D , Yoto Y , Sato T . Ultrasonic assessment of the lingual thyroid gland in children. Pediatr Radiol. 1998;28(2):126‐128. 9472062 10.1007/s002470050311

[5] Ehsan SR , Brandon D , Halkar R . Radioiodine treatment of a symptomatic lingual thyroid. J Nucl Med. 2010;51:1060.

[6] Pellini R , Mercante G , Ruscito P , Cristalli G , Spriano G . Ectopic lingual goiter treated by transoral robotic surgery. Acta Otorhinolaryngol Ital. 2013;33(5):343‐346. 24227901 PMC3825042

[7] Dziegielewski P , Chau JK , Seikaly H , et al. Lingual thyroid in adults: Management algorithm based on swallowing outcomes. J Otolaryngol. 2011;40:19‐26. 21303597

[8] Ersoy Callıoglu E , Bozdemir K , Ulusoy B , Oguzhan T , Korkmaz MH . Lingual thyroid excision with transoral robotic surgery. Case Rep Otolaryngol. 2015;2015:548582. 26064746 10.1155/2015/548582 PMC4439493

[9] Prisman E , Patsias A , Genden EM . Transoral robotic excision of ectopic lingual thyroid: Case series and literature review. Head Neck. 2015;37(8):E88‐E91. 24816912 10.1002/hed.23757

[10] Lauretano AM , Li KK , Caradonna DS , Khosta RK , Fried MP . Anatomic location of the tongue base neurovascular bundle. Laryngoscope. 1997;107(8):1057‐1059. 9261008 10.1097/00005537-199708000-00010

